# Interleukin-1β drives NEDD8 nuclear-to-cytoplasmic translocation, fostering parkin activation via NEDD8 binding to the P-ubiquitin activating site

**DOI:** 10.1186/s12974-019-1669-z

**Published:** 2019-12-27

**Authors:** Meenakshisundaram Balasubramaniam, Paul A. Parcon, Chhanda Bose, Ling Liu, Richard A. Jones, Martin R. Farlow, Robert E. Mrak, Steven W. Barger, W. Sue T. Griffin

**Affiliations:** 1 Department of Geriatrics, University of Arkansas for Medical Sciences, Little Rock, AR 72205 USA; 2 Geriatric Research Education and Clinical Center at the Central Arkansas Healthcare Veterans System, Little Rock, AR 72205 USA; 3 Department of Neurology, Indiana Alzheimer Disease Center, Indiana University, Bloomington, USA; 4 Department of Pathology, University of Toledo Health Sciences Campus, Toledo, OH 43614 USA; 5 Department of Psychiatry, University of Arkansas for Medical Sciences, Little Rock, AR 72205 USA

**Keywords:** IL-1β, GSK3β, PINK1, Alzheimer’s, Parkin, NEDD8, Neddylation, Ubiquitination, Autophagy, Simulation interactions

## Abstract

**Background:**

Neuroinflammation, typified by elevated levels of interleukin-1 (IL-1) α and β, and deficits in proteostasis, characterized by accumulation of polyubiquitinated proteins and other aggregates, are associated with neurodegenerative disease independently and through interactions of the two phenomena. We investigated the influence of IL-1β on ubiquitination via its impact on activation of the E3 ligase parkin by either phosphorylated ubiquitin (P-Ub) or NEDD8.

**Methods:**

Immunohistochemistry and Proximity Ligation Assay were used to assess colocalization of parkin with P-tau or NEDD8 in hippocampus from Alzheimer patients (AD) and controls. IL-1β effects on PINK1, P-Ub, parkin, P-parkin, and GSK3β—as well as phosphorylation of parkin by GSK3β—were assessed in cell cultures by western immunoblot, using two inhibitors and siRNA knockdown to suppress GSK3β. Computer modeling characterized the binding and the effects of P-Ub and NEDD8 on parkin. IL-1α, IL-1β, and parkin gene expression was assessed by RT-PCR in brains of 2- and 17-month-old PD-APP mice and wild-type littermates.

**Results:**

IL-1α, IL-1β, and parkin mRNA levels were higher in PD-APP mice compared with wild-type littermates, and IL-1α-laden glia surrounded parkin- and P-tau-laden neurons in human AD. Such neurons showed a nuclear-to-cytoplasmic translocation of NEDD8 that was mimicked in IL-1β-treated primary neuronal cultures. These cultures also showed higher parkin levels and GSK3β-induced parkin phosphorylation; PINK1 levels were suppressed. In silico simulation predicted that binding of either P-Ub or NEDD8 at a singular position on parkin opens the UBL domain, exposing Ser_65_ for parkin activation.

**Conclusions:**

The promotion of parkin- and NEDD8-mediated ubiquitination by IL-1β is consistent with an acute neuroprotective role. However, accumulations of P-tau and P-Ub and other elements of proteostasis, such as translocated NEDD8, in AD and in response to IL-1β suggest either over-stimulation or a proteostatic failure that may result from chronic IL-1β elevation, easily envisioned considering its early induction in Down’s syndrome and mild cognitive impairment. The findings further link autophagy and neuroinflammation, two important aspects of AD pathogenesis, which have previously been only loosely related.

## Introduction

Neuroinflammation, with its associated elevation of interleukin-1 (IL-1) α and β synthesis and release, and autophagic failure, with its associated buildup of ubiquitinated protein aggregates in autophagosomes, are now recognized as early events in the Alzheimer-related neuropathogenesis of Down’s syndrome [1–4] and, analoguously, of early events in the neuropathogenesis of Alzheimer’s disease (AD) [3, 5, 6]. Such early events culminate in the neuropathological picture of end-stage AD, viz., the accumulation of neuronal and extraneuronal ubiquitinated protein aggregates. Such aggregates certainly include the pathognomonic seed proteins of the amyloid β-peptide (Aβ) present in extraneuronal plaques and the hyperphosphorylated tau present in neurofibrillary tangles in AD, both of which are known to be induced by IL-1β [7–10]. However, it is becoming increasingly clear that other proteins are present in such aggregates consistent with an apparent wholesale collapse of proteostatic mechanisms such as the ubiquitin-proteasome system and autophagy. The myriad connections between autophagy and inflammation, as well as their elaboration during aging, reinforce suppositions about the roles of these processes in age-related neurodegeneration. While neuroinflammation with its excess of cytokines, especially IL-1β, almost certainly evolved (and continues to serve) as an adaptive response to acute neuronal injury and other neural threats, it is widely accepted that chronic neuroinflammation has detrimental consequences. One of those may include interference with proteostasis, thus contributing to the accumulation of aggregates that could themselves serve as additional activators of neuroinflammation.

Autophagy may regulate IL-1β and the development of the above neuropathologies via mechanisms that have been characterized elsewhere [11], but little has been reported concerning a potential role for neuroinflammation and IL-1β in the regulation of specific mechanisms, that may limit the burden of misfolded proteins both prior to and following the formation of aggregates such as those in AD. As background for this report, we have shown in an experimental setting in which glutamate treatment of neuronal cultures was used to induce neuronal stress, the amyloid precursor protein (APP) levels were elevated alongside release of secreted amyloid precursor protein alpha (sAPPα), which, in turn, served as a powerful activator of microglia and of their synthesis and release of IL-1β [12]. From some perspectives, excess IL-1β would seem to be favorable for neuronal recovery from stress. For instance, IL-1β induces neuronal production of specific proteins that are necessary for membrane and synaptic integrity, viz., APP, phosphorylated tau (P-tau), and α-synuclein [10, 13, 14], as well as for microtubule stability and function [15], neurotransmitter deficits [1, 14], and scavenging of fragmented DNA [16]. Generally, these IL-1β-mediated effects would appear to be helpful in maintaining neuronal health and stability. Most studies and reviews of potential interactions between neuroinflammation and autophagy favor the view that increases in autophagy act to balance or modulate neuroinflammatory processes [17, 18]. This is a reasonable supposition as increases in autophagy would enhance clearance of pathogenic aggregates such as Aβ and P-tau, and in this way lessen neuronal stress, and correspondingly decrease microglial activation and IL-1β synthesis and release. However, in AD, there is evidence that IL-1β synthesis remains unabated [19], indicating that IL-β-induced APP and P-tau production is continuing, favoring—as noted in AD pathology—accumulation of Aβ aggregates in extraneuronal plaques and intraneuronal neurofibrillary tangles, which are ubiquitinated and—although present in autophagosomes—remain undigested [20]. With the general notion that innate immune responses in the brain, including those related to neuronal stress, act to some extent to favor recovery, we sought to determine if the inflammatory responses to neuronal stress—especially IL-1β—positively influence mechanisms known to be necessary for autophagic clearance of unwanted proteins and cellular entities that characterize AD and other neurodegenerative diseases.

The activation of parkin, the E3 ubiquitin ligase responsible for tagging unwanted protein for disposal or re-cycling, provides a likely target for testing the influence of IL-1β on autophagy. This is particularly true as parkin wears many hats in maintaining cellular homeostasis, including ubiquitination of unwanted proteins and cellular entities [21]. Parkin activation and the resultant ubiquitination of unwanted entities is most often attributed to the binding of phosphorylated ubiquitin (P-Ub) to parkin [22] and is reported to be neuroprotective in certain circumstances [23, 24]. This suggests that IL-1β elevation could play a similar role in response to acute neuronal stress, and further, if such stress becomes chronic as in AD may contribute to the ample neuropathological evidence of ubiquitination having occurred, perhaps even in excess, in brains of Alzheimer [25] and of Parkinson patients [26, 27]. In either case, parkin activation occurs as a two-step process. Binding of P-Ub results in activation of parkin through exposure of parkin Ser_65_ for phosphorylation [28, 29]. Specifically, binding of P-Ub to parkin allows the auto-inhibitory domain of parkin to free its RING2 (a really interesting new gene) domain, exposing parkin Ser_65_ for phosphorylation [30]. The prevailing evidence is that PINK1 (PTEN-induced putative kinase protein 1) is the kinase that mediates the phosphorylation of both parkin Ser_65_ and ubiquitin Ser_65_ [30, 31], and a decrease in the levels of PINK1 in the brains of Alzheimer patients [32] has been suggested to account, at least in part, for failures in ubiquitination and, thus autophagic clearance in AD. If this is the case, a potential role of neuroinflammation in this process is supported by our finding here that IL-1β reduces synthesis of PINK1.

Neddylation, another parkin-activating process, occurs when NEDD8 (Neural precursor cell -expressed, developmentally downregulated protein 8) is conjugated to parkin and neddylated parkin has been shown to have upregulated ubiquitin ligase activity by enhancing its binding affinity for various ubiquitin-conjugating enzymes [33]. This process appears to be separate and parallel to P-Ub-mediated activation of parkin, which opens parkin’s auto-inhibited form [34]. Despite evidence of multiple connections between IL-1β and NEDD8 [35–37] and other aspects of AD pathology, prior to this report, no detailed model has been proposed for the succession of molecular events leading to parkin activation by NEDD8. In normal neurons, including those in age-matched control (AMC) patients, NEDD8 is most often localized in the nucleus and is associated with its well-characterized family of substrates, the Cullin-RING family of E3 ubiquitin ligases [38], which function to suppress DNA replication, cell cycle re-entry, and cell death [39]. However, in contrast to neurons in AMC patients, nuclei of neurons in patients with AD are relatively deficient in NEDD8 [40], which appears to have translocated to the cytoplasm [41]. Moreover, NEDD8 also accumulates in aggregated inclusion bodies in neuronal cytoplasm of patients with Parkinson’s, amyotrophic lateral sclerosis, and dementia with Lewy bodies [42], much as it does in AD. Until the present study in which we provide evidence of such translocation of NEDD8 in neuron cultures treated with IL-1β, neither the triggering mechanism(s) by which translocation of NEDD8 from nucleus-to-cytoplasm occurs nor the events that foster activation of parkin by NEDD8 have been elucidated. Our results may explain, at least in part, how neuronal stress-induced excessive levels of IL-1β may act as a driver of ubiquitination early in AD pathogenesis through induction of APP expression [13, 14].

## Methods

### Human hippocampal tissues

Tissue samples were acquired at autopsy at the University of Arkansas for Medical Sciences (UAMS) and were neuropathologically evaluated by one of us (REM). Patients designated as age-matched controls (AMC) were clinically described as non-demented, and were evaluated, according to the CERAD (consortium to establish a registry for Alzheimer’s disease) criteria as lacking AD pathology, and assessed by Braak staging as 1 or 0 [43, 44]. All AD cases were given CERAD scores of C (definite Alzheimer’s), and Braak stages IV-VI. Samples were stored in our UAMS Brain Bank either as snap-frozen 3-D dissected regions or as formalin-fixed, paraffin-embedded blocks. Tissue from a total of 6 AD and 5 AMC patients was used in these experiments. The average age of the patients was 76, with post-mortem intervals (PMI) between 3 and 13 h, with an average PMI of 5.3 h.

### Animals

Pregnant female Sprague-Dawley rats were obtained from Jackson Laboratories, Bar Harbor, ME, USA, as the source for 18-day-old fetuses for primary neural cell culture experiments. Pregnant females were given ad libitum access to tap water and chow (Harlan Teklad, Madison, WI, USA) and were housed at an ambient temperature in our AALAC-approved facility at the Veterans Administration Medical Unit (VAMU). The mice used in this study were raised to ages 2 or 17 months under standard conditions (25 °C) and offered commercial mouse chow and tap water ad libitum. The experimental group was designated Strain 109 by Games et al. [45] and consisted of 6 homozygous female transgenic PD-APP (hAPP v717f) littermates, aged either two or 17 months for a total of 12, and the control group consisted of 12 wild-type littermates, 6 were 2 months and 6 were 17 months old; all such mice are available at Taconic Farms, Inc. (Germantown, NY). After ketamine overdose, the left cerebral cortex was flash frozen in preparation for RT-PCR experiments.

### Ethics, consent, and permissions

Human tissues used for this study were collected at autopsy for reasons other than for this study and were judged as exempt from Federal regulations on human subjects via exemption 4, with Human Subject Assurance Number 00001119 by the University in strict accordance with the recommendations of US National Institutes of Health Guide for the Care and Use of Laboratory Animals. All protocols were approved (approval number, a3063-01) by the Institutional Animal Care and Use Committee (IACUC) and all efforts were made to minimize pain and suffering.

### Cell cultures and treatment

#### Primary cells

Primary neuronal cells were harvested as previously described [46]. In brief, cells were harvested from cerebral cortex of gestational day 18 Sprague-Dawley rats, and 5 × 10^5^ cells were plated in poly-D-lysine-coated 35-mm plates and maintained in neurobasal medium (Fisher, Pittsburgh, PA). After 10 days, medium was removed and fresh medium was added to the neurons. After 24 h, cells were either treated overnight with 30 ng/mL IL-1β, or left untreated in 1% fetal bovine serum (FBS) in DMEM. Medium was then removed, and cells were washed with 1×PBS, protein extracted in 1×RIPA buffer. For immunocytochemistry, cells were fixed in 4% paraformaldehyde for 1 h at RT, rinsed, and stored at 4 °C until used.

#### NT2 cells

Cells (NT2, aka NTERA2/D1, from ATCC) were grown in 75 cm^2^ flasks containing 12 mL DMEM and 10% FBS. Flasks were placed in an incubator maintained at 37 °C in a 5% CO_2_–95% air atmosphere. For experiments with IL-1 β, confluent cells were separated by treatment with 0.125% trypsin and 1 mM EDTA. On day 0, 1 × 10^6^ cells were plated onto 35-mm culture dishes, and 0.5 × 10^3^ cells were plated in 4 well chamber slides, or on cover slips in DMEM containing 10% FBS. When the cells were attached to the culture dishes, medium was changed to DMEM containing 1% FBS, and cells were either treated with 30 ng/mL of IL-1β (Sigma, St. Louis, MO) or left untreated (control) overnight. After treatment, cells were washed with 1× cold PBS and used for the following studies. Each experiment was repeated at least six times.

#### Nuclear and cytoplasmic extraction

NT2 cells were plated and treated as above. For cytoplasmic and nuclear fractionation, NE-PER™ Nuclear and Cytoplasmic Extraction Reagents (CER) kits (ThermoFisher Scientific) were used as per manufacturer’s directions. In brief, after treatment, medium was removed and cells were washed with cold 1×PBS, harvested with trypsin-EDTA, and centrifuged at 500×*g* for 5 min. Cell pellets were washed by suspending the cells in 1×PBS. Cells were then transferred to 1.5-mL tubes and centrifuged at 500×*g* for 3 min and supernatants were discarded, leaving the cell pellet as dry as possible. Ice-cold CER I buffer was added to the cell pellets, and tubes were vigorously vortexed on the highest setting for 15 s to fully suspend the cell pellet. Tubes were kept on ice for 20 min, then ice-cold CER II was added to the tubes, which then were vortexed as before, incubated on ice for 5 min, vortexed on the highest setting for 5 s, and centrifuged at 16,000×*g* for 5 min. Supernatants, i.e., the cytoplasmic extracts, were transferred to pre-chilled tubes. For the nuclear fraction, insoluble pellets were suspended in ice-cold nuclear extraction reagent (NER) then vortexed at the highest setting for 15 s. Samples were placed on ice for 40 min, vortexed for 15 s at 10-min intervals. Tubes were then centrifuged at 16,000×*g* for 10 min. Supernatants, i.e., the nuclear extract, were transferred to pre-chilled tubes. Protein was quantified using a Micro BCA assay reagent kit (Pierce, Rockford IL). Samples were stored at − 80 °C until use.

#### Western blot analysis

For western analysis of protein, cells were lysed with 1×RIPA buffer (20 mM Tris HCl (pH 7.5), 150 mM NaCl, 1% Nonidet P40, 1 mM EGTA, 1 mM EDTA, and 1% sodium deoxycholate), and total protein was quantified using a Micro BCA assay reagent kit. Equal amounts of total protein (30 μg/lane) were loaded onto a 4–12% Tris-glycine precast gel (Invitrogen Life Technologies; Carlsbad, CA). For NEDD8, nuclear and cytoplasmic fractions were subjected to electrophoresis on 4–12% bis-Tris gels in MES buffer (Invitrogen Life Technologies), and fractionated protein was transferred to nitrocellulose membranes at 30 V for 1 h at room temperature. Membranes were blocked in 5% non-fat dry milk in 1×TBS for 1 h at room temperature and then probed with primary antibodies: anti-NEDD8 rabbit polyclonal (Invitrogen, 34-1400); anti-parkin rabbit mAb (Abcam ab179812); anti-P-parkin(Ser_65_) sheep pAb (Cosmo Bio, CA, 68-0056-100); mouse mAb PINK-1 (Abcam, ab186303), for NT2 cells; and Stress Marq (SMC-450), for rat primary neurons; anti-P-ubiquitin (Ser65) rabbit mAb (Cell Signaling Technology, 37642) diluted to 1:1000 in 5% non-fat dry milk; and for phosphospecific antibodies in 5% BSA in 1× TBST; 20 mmol/L Tris HCl, 137 mmol/L NaCl, and 0.2% (vol/vol) Tween 20 (pH 7.6 TBST), and incubated overnight at 4 °C with gentle shaking. Membranes were washed five times (5 min each) with TBST and incubated with horseradish-peroxidase coupled anti-IgG for 1 h at room temperature. The secondary antibody was diluted 1:2000 for anti-rabbit (Cell Signaling Technology), anti-mouse, and anti-sheep (R&D Systems). Membranes were stripped and re-probed with actin/GAPDH Rb actin and mouse GAPDH, (Santa Cruz Biotechnology) for identigication of the cytoplasmic fraction, and Histone H1 for nuclear compartment identification, and as a test of equal loading and normalization. For inhibitor studies, cells were pretreated with 5 or 30 μM of GSK3β (Glycogen synthase kinase-3 beta inhibitor) (either XXVII-Calbiochem (cat#361570) or AR-A 014418, Tocris Biosciences (cat#3966) for 4–6 h before adding 30 ng/mL IL-1β for 16 h. Cells were collected and total protein lysates were prepared and processed for western immunoblot asessment of phosphorylated parkin (P-parkin) and P-ub levels. Non-phospho-parkin peptide (Cat# 68-1010-001) was added to the antibody incubation in order to deplete any non-phospho specific parkin. For visualization of the bands, Enhanced Chemiluminescence (Super-Signal West Pico Chemiluminescent Substrate; Thermo Scientific, Rockford, IL) was used. Bands were quantified using an Alpha Innotech ChemiImager 5500 (Alpha Innotech Corporation, Santa Clara, CA).

#### Lipofection

For the GSK3β siRNA transfection study, target-specific 20–25 nucleotide small interfering RNAs (siRNAs) for GSK3β was commercially obtained (Sigma; product no. NM_002093) and transfected by lipofection using Lipofectamine RNAiMAX (Invitrogen; catalog no. 13778-150). The Lipofection protocol was followed as per manufacturer’s protocol. In brief, NT2 cells (2 × 10^6^ cells/plate) were seeded in 100-mm tissue culture plates in 10 mL antibiotic-free DMEM containing 10% FBS. Cells were incubated at 37 °C overnight in an incubator with 5% CO_2_ in air. After 24 h, cells were washed with 2 mL transfection medium, and a mixture of 50–80 pmol of siRNA, and transfection reagent (lipofectamine RNAiMAX) diluted in siRNA transfection medium was added to the washed cells. Cells were incubated for 24 h at 37 °C in the incubator. After 24 h of incubation, the medium was replaced with 1× normal growth medium, and cells were treated with 30 ng/mL IL-1β, and cells were further incubated for 16 h after which the cells were harvested and P-parkin levels were assessed by western immunoblot.

#### Immunocytochemistry

After IL-1β treatment, cells were washed with 1× cold PBS and fixed with 4% neutral buffer formaldehyde for 30 min at room temperature, after washing with 1×PBS three times for 5 min each, and then rinsed with 100% alcohol. Non-specific sites were blocked by incubating the cells in 0.1% triton x-100 and 5% goat serum for 30 min at room temperature and washed 3 times with 1×PBS for 5 min each. Cells were incubated with 2–10 μg/mL primary antibodies, rabbit polyclonal NEDD8 (Invitrogen) diluted in 1% BSA and 1×PBS at 37 °C for 1 h. Cells were washed 3 times with 1×PBS for 5 min each. FITC (fluorescein isothiocyanate; Abcam) conjugated secondary antibody, 2–10 μg/mL, diluted with 5% goat serum in 1×PBS, was added to the cells and incubated at 37 °C for 1 h. For negative controls, cells were incubated with IgG only, without specific primary antibodies. Cells were washed 5 times with 1×PBS for 5 min each and mounted with Vectashield mounting media (Vector Laboratories Inc. Burlingame, CA) containing DAPI for counterstaining the nuclei.

#### Human hippocampal immunohistochemistry

Hippocampal tissue blocks were sectioned at 7-μm thickness and deparaffinized in xylene, rehydrated in serial dilutions of ethanol to water, and washed with PBS + 0.1% Tween 20. Endogenous peroxidase activity was blocked with 3% H_2_O_2_. Antigen retrieval was performed in citrate buffer (100 °C for 30 min) and the endogenous background activities were blocked in normal rabbit serum according to instructions in the Vector Elite Goat Kit (PK-6105) for 20 min, then incubated in primary antibody parkin (R&D AF1438) at 1:250 overnight at 4 °C. The sections were incubated in biotinylated antibody for 30 min, and then in the ABC complex for 30 min, followed by 3 min in DAB chromagen. After serial dehydration, the sections were cover-slipped with Micromount. For double staining with IL-1α (sc-1279, Santa Cruz Biotechnology) and parkin, endogenous background was blocked with 100% normal horse serum for 30 min at RT. Sections were incubated in IL-1α antibody at 1:200 overnight at 4 °C. After washing, sections were incubated in goat ImmPRESS peroxidase reagent for 30 min followed by ImmPACT DAB solution for 3 min. After washing with water to PBS-Tween 20, sections were incubated with parkin antibody (1:100) overnight in 4 °C. After primary antibody incubation and washing, sections were incubated in rabbit ImmPRESS peroxidase reagent for 30 min followed by a 2-min incubation with Deep Space Black Chromagen solution (BioCare Medical, Concord, CA) to quench lipofuscin interference. After serial dehydration, the sections were cover slipped with Micromount.

#### Proximity ligation assay

Proximity ligation assay (PLA) is a specialized molecular technique which can probe molecular colocalization in fixed tissue with much greater specificity than other current methods. In brief, PLA produces a fluorescent signal (dot) when two molecules probed by primary antibodies are within 400 Å of each other. Duolink PLA was performed according to the manufacturer’s protocol (Sigma, St. Louis, MO). Procedures described above for immunohistochemistry (IHC) or immunocytochemistry (ICC) were performed until the primary antibody incubation step. Subsequent to this, samples were treated with oligonucleotide-conjugated secondary antibodies (DUO92002 anti-rabbit PLUS and DUO92004 anti-mouse MINUS) for 1 h at 37 °C, washed in Duolink Wash Buffer A, incubated for 30 min with Duolink ligation solution at 37 °C, and washed again in Wash Buffer A. Then, slides were incubated with Duolink DNA polymerase solution for 100 min at 37 °C for amplification of the PLA signal. Autofluorescence was quenched in 0.1% Sudan Black B in 70% EtOH for 2 min. Slides were mounted in Duolink Mounting Medium with DAPI and sealed with clear nail polish.

#### RT-PCR amplification

Total RNA was extracted from brain tissue using TriReagent™ RNA (Molecular Research Center, Cincinnati, OH), according to the manufacturer’s instructions. Real-time polymerase chain reaction (RT-PCR) was performed as previously described [47]. Briefly, for comparisons of mRNA levels among different RNA samples, RT reactions were performed simultaneously using SyberGreen Master Mix reagents from Life Technologies (Grand Island, NY). The sequences of primers for IL-1α, IL-1β, and parkin are as follows: IL-1α-F 5′-CCC AGA TCA GCA CCT TAC ACC TA-3′; IL-1α-R IL-1a R 5′-CTT CTG CCT GAC GAG CTT CAT-3′; IL-1β-F IL-1β F 5′-TGG TGT GTG ACG TTC CCA TT-3′; IL-1β-R 5′-CAG CAC GAG GCT TTT TTG TTG-3′; parkin-R 5′-ATC ACT GGT TTG ACG TGT AGA G-3′; parkin-F 5′-AAG AAA TGG CTA AGG AAG GTA GG-3′. PCR conditions were 40 cycles at 95 °C for 10 min, 95 °C for 15 s, and 60 °C for 1 min. Equal amounts of samples were pooled to use for standard curve development with each primer set to verify linearity and a suitable slope. All given mRNA tissue levels are relative to 18 s RNA.

#### Image analysis

Images were taken on a Nikon Eclipse E600 Microscope with a CoolSnap ES camera. Quantitative immunofluorescence was performed with the same exposure times among all samples for the same measurement.

#### Human brain

A total of 5 AMC and 6 AD patients were used for IHC analysis of nuclear NEDD8, and five images were taken of each patient slide in the CA1 area of the hippocampus in the case of human data. Proximity ligation assay (PLA) experiments were taken from a total of six patient slides, with 5 images per slide. The average number of cells per image was 38. Total number of cells counted was 1146: 615 in AMC and 531 in AD.

#### Cell culture

A total of 6 slides per group were used for analysis of IHC and five images were taken, per slide, in an operator-blinded manner. Nuclear NEDD8 was measured via an automated process in which DAPI was used as a stencil to outline nuclei, and fluorescence intensity values were measured within these nuclei, which was then divided by the nuclear area. Quantitative analyses were done by Image J, version 1.46 software.

#### Predictive structure modeling

Because the available full-length crystal structure of human parkin contains either mutated residues or deletions, we modeled the native full-length structure of parkin, using comparative modeling techniques with Modeller9V8; Ser_65_ was modified to phospho-serine using VIENNA PTM. NEDD8 and ubiquitin structures were retrieved from PDB (2N7K and 5CAW respectively). All structures that included this modeled parkin were energy minimized in a solvent system using the GROMACS simulation package.

#### Protein-protein docking

Protein-protein interactions were calculated using Hex8.0 (Linux version). The correlation type was set to shape plus electrostatics, and post-processing was set for OPLS minimization. Other parameters, including receptor-ligand distances, were set to default. Out of 2000 possible orientations, minimum energy conformer was used for further analysis. To perform the second docking, the parkin-NEDD8 complex, which was the result of the first docking, was energy minimized in the implicit solvent method, using the Discovery Studio Simulation Package. This energy-minimized structure was then used for calculating the secondary docking of parkin-NEDD8 with UbcH8, using the parameters mentioned above.

#### Atomistic metadynamics simulation

For metadynamics simulation, the docked structures were prepared using standard protein preparation wizard from Maestro 2018-1 suite. Simulation system was prepared using System Builder wizard. The protein complex was centered in an orthorhombic box, 10 Å away from all edges of the simulation box. Solvation was completed by adding simple-point-charge (SPC) water after which the system was neutralized with NaCl as counter ions. In order to maintain the physiological pH, an additional 0.15 M NaCl was added to the simulation box. The solvated system was then equilibrated and simulation was conducted using a metadynamics approach, using GPU accelerated Desmond v2018-1 [48]. The collective variable (CV) for all the metadynamics runs mentioned in this work includes distance between Val_70_ and Glu_310_ of the parkin structure. Other CV parameters were kept as default. Simulations were performed for 100 ns using the RESPA integrator. The results were analyzed in Maestro visualizer.

#### Statistical analysis

All analyses were performed using Prism 6.0 (GraphPad Software Inc., San Diego, CA). For normally distributed data, unpaired one-way analysis of variance (ANOVA) with Tukey-Kramer multiple comparisons test was used. Data from at least three independent experiments were used for statistical analyses. Data presented here are given as mean ± SEM of each triplicate. Post hoc analysis with unpaired two-tailed Student *t* tests were used when comparing means between two groups. An unadjusted *p* value of equal to or less than 0.05 (*p* ≤ 0.05) was considered significant. PLA studies for human tissue, with *n* = 3 per group, were measured with the non-parametric Wilcoxon Rank Sum Test for significance, *α* = 0.05.

## Results

We present here the first evidence that the neuroinflammatory mediator IL-1β facilitates ubiquitin ligase parkin/NEDD8 interactions as it: (i) elevates the steady-state levels of parkin and phosphorylated parkin; (ii) induces nuclear-to-cytoplasmic translocation of NEDD8; and (iii) induces NEDD8-parkin ligation in the cytoplasm of hippocampal pyramidal neurons in AD. Moreover, our molecular modeling predicts that activation of parkin by NEDD8 is the result of NEDD8 binding to parkin at the same site that Ser_65_-phosphorylated ubiquitin (P-Ub) uses for its activation of parkin, suggesting that activation of parkin by either P-Ub or NEDD8 may serve as a necessary step in parkin-mediated ubiquitination of unwanted entities designated for lysosomal degradation.

### Experimental evidence that regulation of parkin expression and phosphorylation are related to glial activation and excess IL-1β in Alzheimer brain and in AD Model Systems

**Fig. 1:**
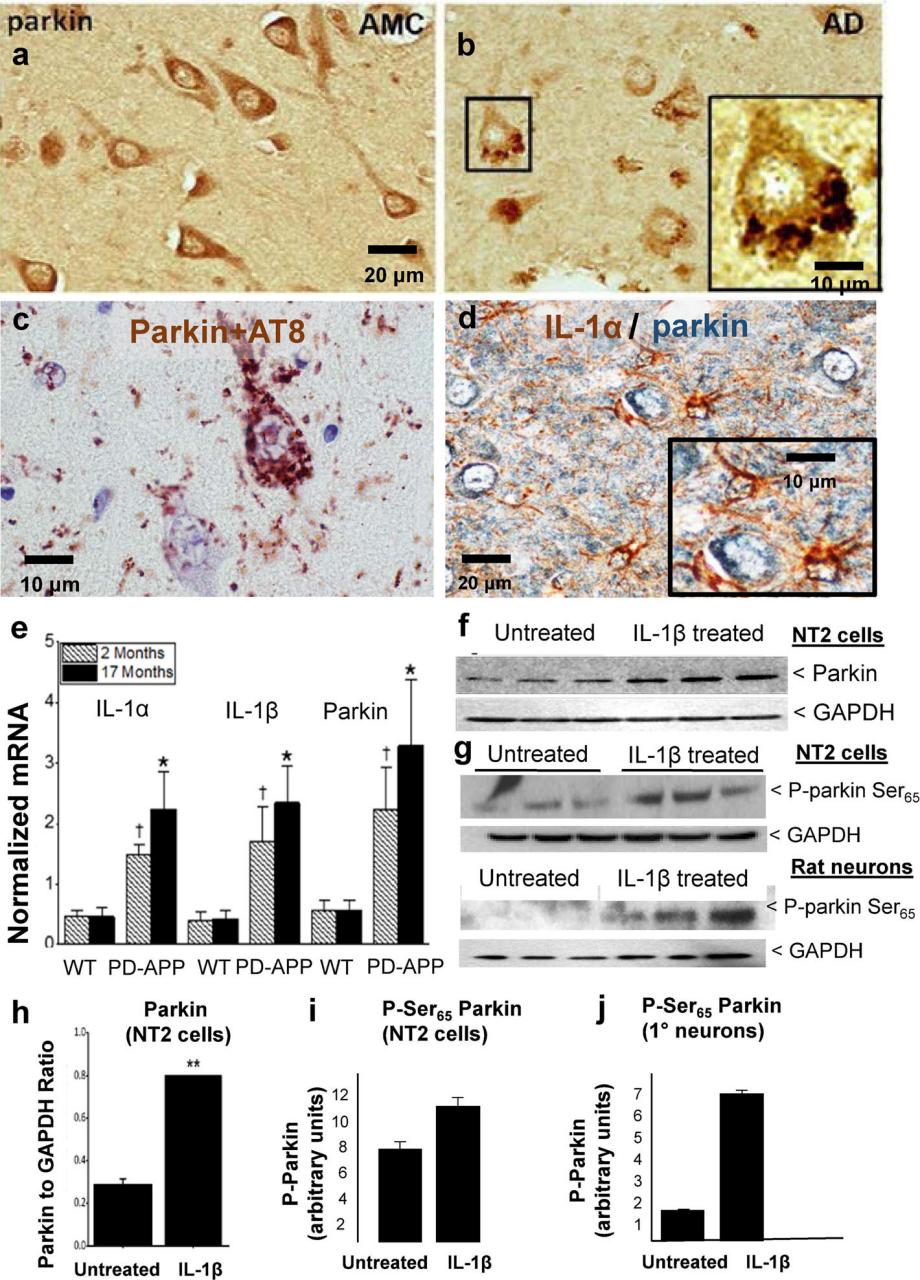
Experimental evidence that regulation of parkin expression and phosphorylation is related to glial activation and excess IL-1β in Alzheimer brain and in AD Model Systems. **a** Parkin in neurons in brains of age-matched control patients (AMC) is diffusely distributed within the cytoplasm whereas parkin in neurons in brains of Alzheimer patients (AD) is present in rosette-like aggregates (**b**). **c** Parkin in Alzheimer brain is colocalized with tau aggregates. **d** Activated glia overexpressing IL-1α (brown) are immediately adjacent to parkin-immunoreactive neurons (blue), scale bar 20 μM. **e** Comparative levels of IL-1α, IL-1β, and parkin mRNAs in wild-type littermates or PD-APP mice. **f** Representative western immunoblot showing proteins from NT2 cells either treated, or not, overnight with IL-1β. **g** Phospho-parkin levels (P-parkin Ser_65_) in two representative western immunoblots of proteins from NT2 cell and rat primary neuron cultures treated, or not, with IL-1β. **h** Quantification of parkin in untreated and treated cell cultures (*n* = 6), data is mean ± SEM (**p < 0.01). **i**, **j** Quantification of P-parkin levels in IL-1β treated or untreated cultures in NT2 cells (**i**) and primary rat neurons (**j**)

In contrast to the homogenous distribution of parkin in pyramidal hippocampal neurons of control patients (Fig. 1a), we observed parkin in tightly packed rosettes in analogous neurons in hippocampus of Alzheimer brains (Fig. 1b) and showed by proximity ligation assay (PLA) that the colocalization of this parkin and AT8-immunoreactive tau (P-tau) is very tight, i.e., within 400 Å in these neurons (Fig. 1c). Further, we found that these parkin-P-tau-laden neurons were surrounded by IL-1α-overexpressing glial cells (Fig. 1d, Parkin in blue, IL-1α in brown). As a follow-up, in the PD-APP mouse model of Alzheimer, we found that the synthesis of IL-1α, IL-1β, and parkin was elevated in mice as early as 2 months of age (Fig. 1e, *p* = 0.036 for IL-1α, *p* = 0.04 for IL-1β, and *p* = 0.030 for parkin), months before Alzheimer lesions have been noted in this model [49]. Moreover, these differences intensified as the mice aged to 17 months (IL-1α, *p* = 0.015; IL-1β, *p* = 0.014; parkin, *p* = 0.028); wild-type littermates of the PD-APP mice showed no elevation in the levels of any of the mRNA products measured to indicate IL-1α, IL-1β, and parkin synthesis, age-related or otherwise. In a test of the potential of IL-1β to regulate parkin expression, NT2 cells treated with IL-1β overnight were found to have an increase in the steady-state levels of parkin (Fig. 1f and h, *p* < 0.01), as well as in Ser_65_-phosphorylated parkin (Fig. 1g, i, j), indicating that IL-1β acts to increase activity of parkin through both increasing parkin expression as well as its phosphorylation.

### IL-1β treatment of both NT2 cells and rat primary neurons decreased the steady-state levels of PINK1 and of Ser_65_-phosphorylated ubiquitin (P-Ub); conversely, IL-1β increased the levels of Ser_65_-phosphorylated parkin (P-parkin)

**Fig. 2:**
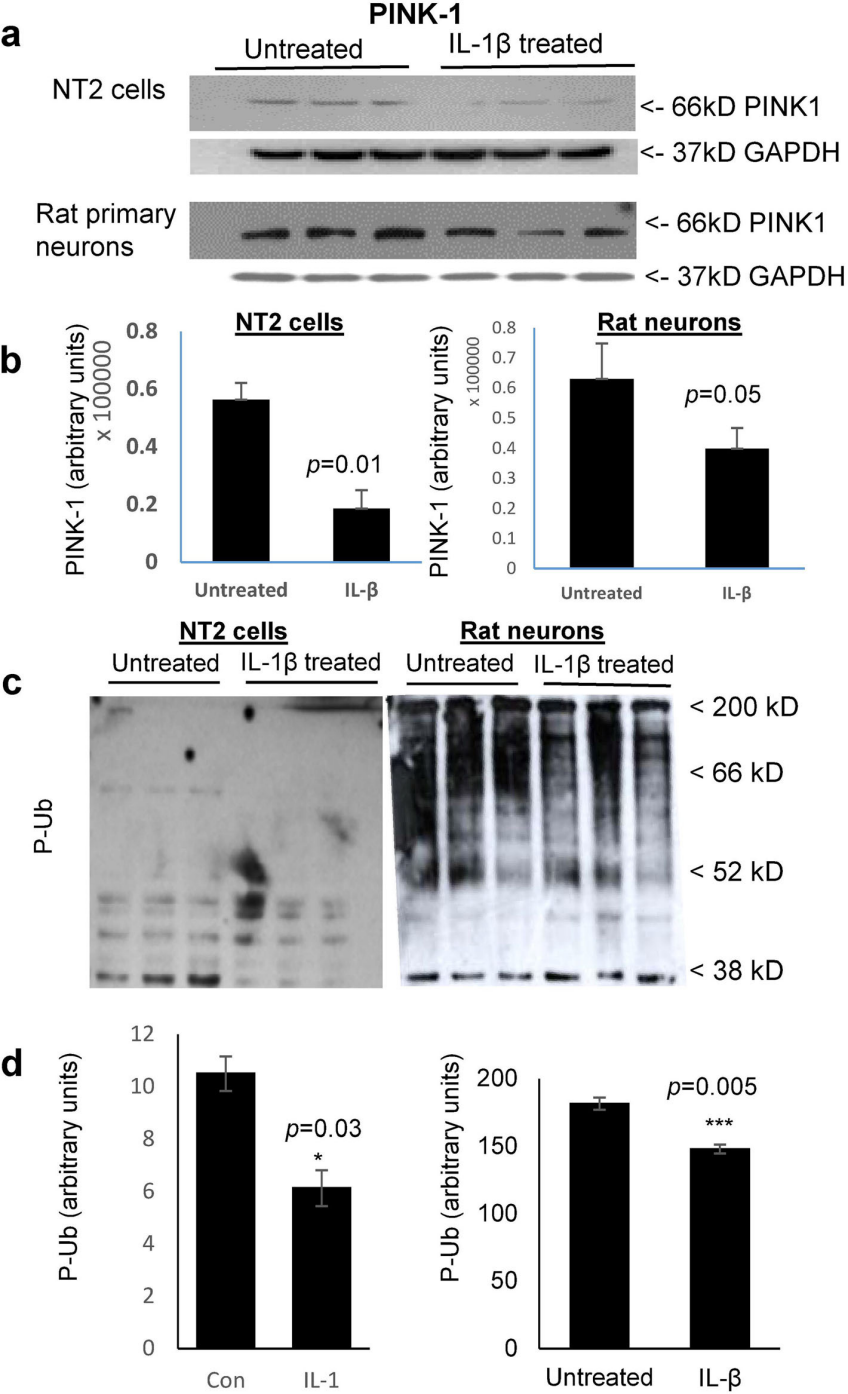
IL-1β treatment of both NT2 cells and rat primary neurons decreased the steady-state levels of PINK1 and of Ser_65_-phosphorylated ubiquitin (P-Ub); conversely, IL-1β increased the levels of Ser_65_-phosphorylated parkin (P-parkin). **a** Representative western immunoblots depicting PINK1 levels in IL-1β treated vs untreated NT2 and rat primary neuronal cell cultures, representing 6 independent experiments. **b** Quantification of PINK1 levels in both NT2 cells (*p* = 0.01), and primary rat neurons (*p* = 0.05). **c** Representative western immunoblots of IL-1β treated vs untreated NT2 and rat primary neuronal cell cultures (*n* = 6 and 3 technical repeats, respectively). **d** Quantification of P-Ub levels in both NT2 (*p* = 0.03) and rat primary neurons (*p* = 0.005)

Our finding of an IL-1β-mediated increase in the steady-state levels of both parkin and Ser_65_-phosphorylated parkin was surprising as IL-1β treatment downregulated the levels of PINK1 (Fig. 2a, b), which is the kinase reported to be responsible for phosphorylation of both parkin and its activator P-Ub. Interestingly, our finding of IL-1β suppression of PINK1 levels in NT2 cell and primary neuronal cultures is in accord with the lower levels of PINK1 noted in Alzheimer brain relative to that in the brain of age-matched controls [32]. Consistent with this downregulation of PINK1, we also saw a concomitant decrease in P-Ser_65_ Ubiquitin (Fig. 2c, d). This is important as decreased PINK1 signaling would be expected to result in a decrease in both Ser_65_-P-Ub and subsequent opening of the parkin ubiquitin-like domain (UBL domain) for exposure and phosphorylation of parkin Ser_65_, which is a necessary step for parkin activation [29]. However, although the levels of P-Ub were less in IL-1β-treated cells, the levels of phosphorylated parkin were elevated in these same cultures. It was this conundrum that led us to investigate the previously reported potential of NEDD8 to activate parkin [33], though the exact mechanism through which NEDD8 accomplishes this is not clearly elucidated.

### Cytoplasmic NEDD8 levels are higher in hippocampal pyramidal neurons in Alzheimer brain than in analogous neurons in control brains, and parkin and NEDD8 are colocalized in a disease-specific manner

**Fig. 3:**
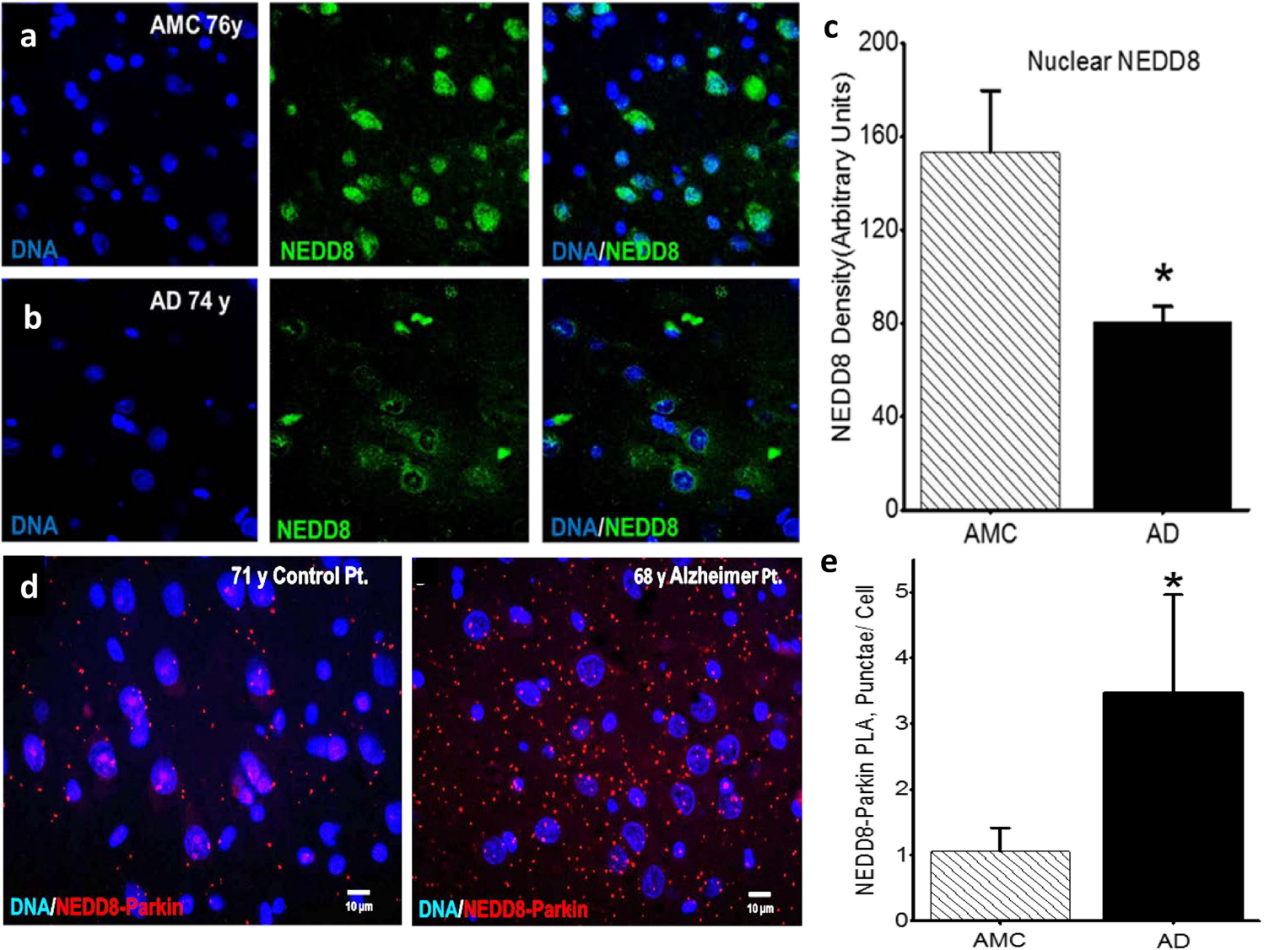
Cytoplasmic NEDD8 levels are higher in hippocampal pyramidal neurons in Alzheimer brain than in analogous neurons in control brains, and parkin and NEDD8 are colocalized in a disease-specific manner. **a** Compared with their age-matched control (AMC) counterparts (*n* = 5), **b** nuclei (blue) of neurons in Alzheimer patients (*n* = 6) have less NEDD8 (green) (**p < 0.005) (**c**). **d** Moreover, in representative images of hippocampal pyramidal cells from these AMC and AD patients (**e**), colocalization of NEDD-8/Parkin (red dots depicted by PLA represent proximity of ≤ 40 nm) is less in AMC than in analogous neurons in Alzheimer patients *p < 0.05 on Wilcoxon rank sum test, *n* = 3 per group

Based on the apparent failure of adequate neuronal clearance of aggregates in AD, together with the known translocation of NEDD8 from nucleus-to-cytoplasm in pyramidal neurons in Alzheimer brains [41], we characterized, confirmed, and quantified such NEDD8 translocation toward understanding the reported NEDD8-related increase in parkin activity [33]. Preferential translocation of NEDD8 from the nucleus to the cytoplasm in AD brain is illustrated in Fig. 3a and b, and quantified in Fig. 3c, *p* < 0.005. As parkin was elevated in our Alzheimer mouse model, and NEDD8 is preferentially localized to the cytoplasm in AD, we were interested to know whether elevated cytoplasmic NEDD8 is colocalized with parkin, suggestive of elevated parkin-NEDD8 interaction in Alzheimer brain. Using PLA, we found that in all age-matched controls, the number of parkin-NEDD8 colocalized dots was less in comparison with their Alzheimer counterparts (Fig. 2d, f, *p* = 0.05, Wilcoxon rank sum test), suggesting that in response to the neuronal stress of AD, NEDD8 translocation occurs alongside elevated parkin and parkin-NEDD8 interaction, perhaps at the behest of elevated IL-1β.

### IL-1β treatment of primary neuronal cultures leads to NEDD8 translocation

**Fig. 4:**
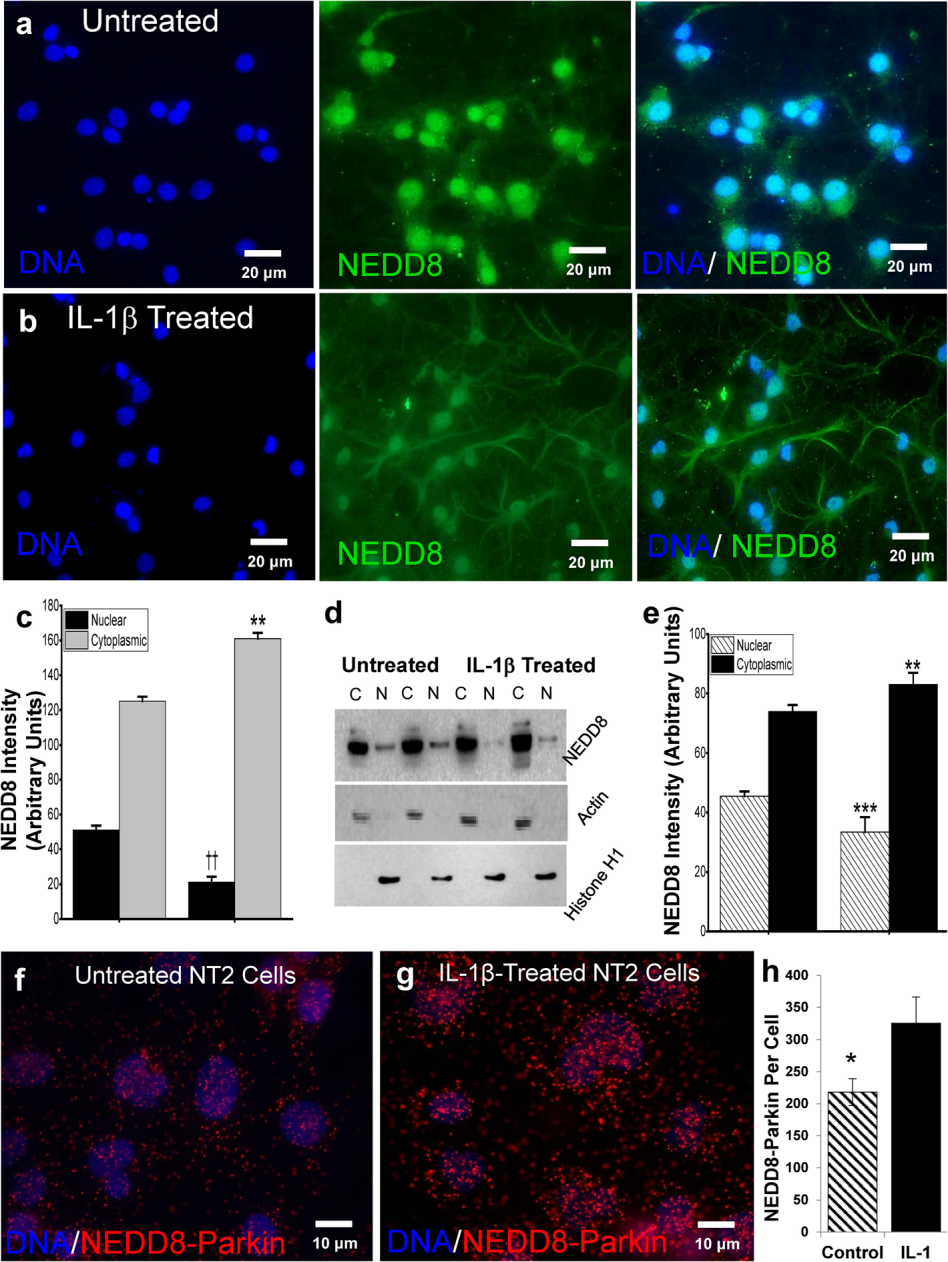
IL-1β treatment of primary neuronal cultures leads to NEDD8 translocation. Fluorescent immunocytochemistry of representative cultures of untreated (**a**) and IL-1β-treated (**b**) primary rat neurons (*n* = 6 each) depict greater translocation of NEDD8 (green) from nucleus (blue) to cytoplasm when IL-1β is present, **p < 0.01, ^††^*p* < 0.01 (**c**). Quantification of western immunoblots (**d**) of nuclear (histone) and cytoplasmic (actin) cell fractions from untreated and IL-1β-treated cultures (*n* = 6 separate experiments) demonstrates the susceptibility of NEDD8 to undergo nuclear to cytoplasmic translocation in the presence of excess IL-1β, **p < 0.01, ***p < 0.01 (**e**). Quantification of NEDD8/parkin colocation (≤ 40 nm) in untreated NT2 cells (**f**) compared with IL-1β-treated NT2 cells (**g**) provides evidence of IL-1β-induced interaction between of parkin and NEDD8 in NT2 cells, two-tailed *t* test, *p < 0.05 (**h**). NEDD8-parkin proximity (red dots) was quantified with FIJI (ImageJ) Particle Analysis

Although the steady-state levels of NEDD8 were similar, rat primary neurons treated with IL-1β showed elevated cytoplasmic NEDD8 and decreased nuclear NEDD8 by immunocytochemistry (Fig. 4a–c, *p* < 0.01). western blot analysis of cytoplasmic and nuclear fractions from IL-1β treated and untreated NT2 cells provided further evidence of such translocation (Fig. 4d, e; nuclear fraction normalized to histone H1, *p* < 0.001; cytoplasmic fractions normalized to actin, *p* < 0.01), demonstrating that IL-1β can effect NEDD8 translocation from nucleus-to-cytoplasm. As to whether this translocation results in parkin-NEDD8 interaction, we found by PLA that overnight treatment of neuronal cultures with IL-1β resulted in elevation of NEDD8-parkin colocalization, (Fig. 4f–h, *p* = 0.048). This data is consistent with the idea that neuroinflammation, in particular its associated elevation of IL-1β, contributes to the translocation of NEDD8, perhaps in an attempt to activate parkin toward ubiquitination of unwanted proteins and aggregates and, thus, lysosomal degradation of aggregates.

### Molecular modeling indicates NEDD8 binds to parkin in the same position as P-Ub and opens up the UBL domain in a manner similar to P-Ub

#### Phosphorylated ubiquitin binding and activation of parkin

**Fig. 5:**
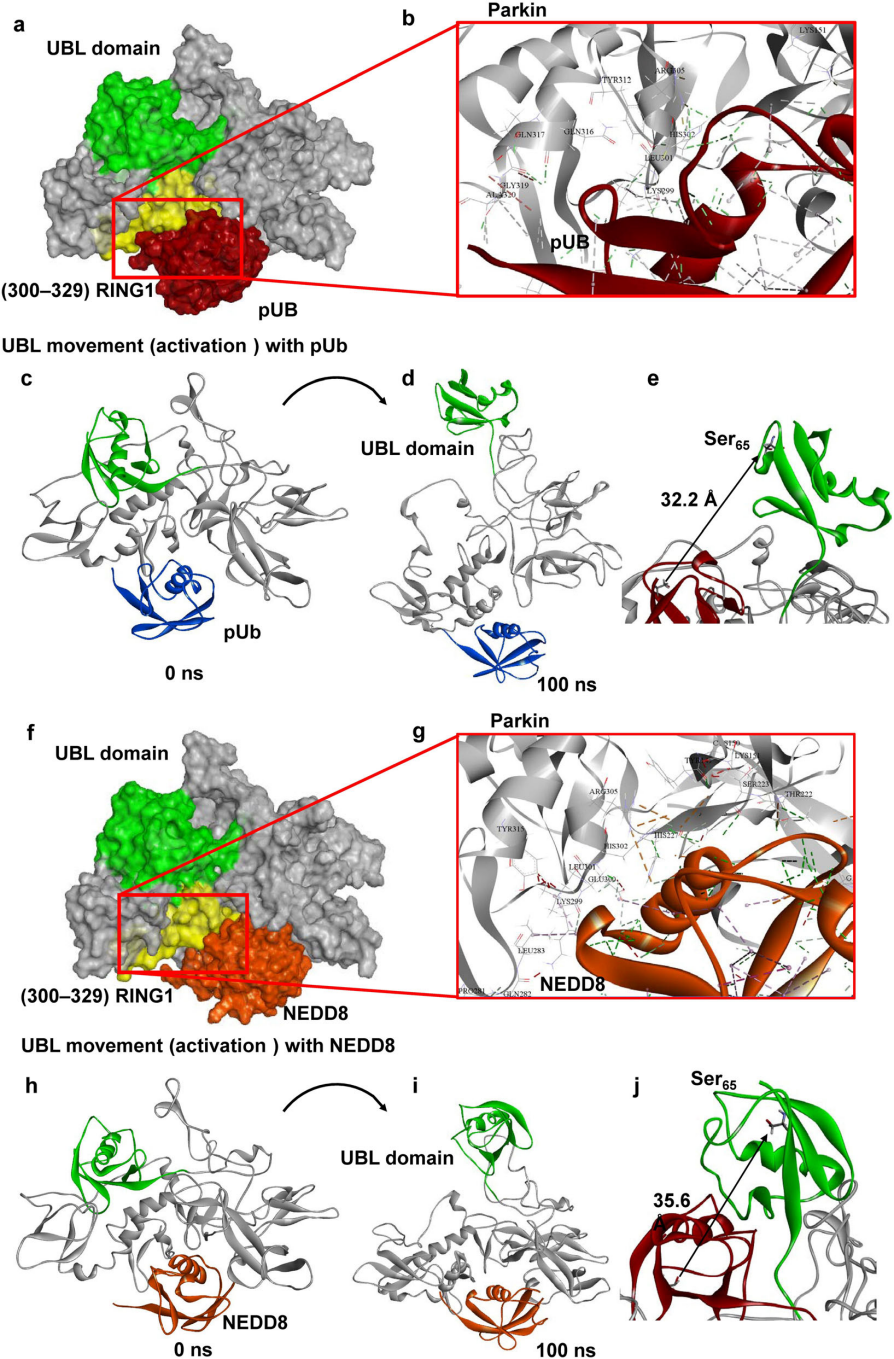
Molecular modeling indicates NEDD8 binds to parkin in the same position as P-Ub and opens up the UBL domain in a manner similar to P-Ub**. a** Molecular interactions for P-Ub with parkin at the P-Ub binding helix agree with the previous experimental model of P-Ub interaction with parkin [1] (**b**). All-atom metadynamics simulation of the P-Ub-parkin complex predicts the movement of the UBL domain exposing the Ser_65_ from the auto-inhibited form of parkin (**c–e**). Protein-protein docking predicts NEDD8 interacts with the auto-inhibited form of parkin similar to that of P-Ub (**f**). Molecular interaction analysis indicates that NEDD8 binds to parkin at the same location as P-Ub, i.e., at the P-Ub binding helix (**g**). All-atom metadynamics simulation of the NEDD8-parkin complex, like that of P-Ub, also opened the UBL domain, exposing Ser_65_ (**h–j**)

Cytosolic parkin usually exists in a “closed” conformation, in which parkin is auto-inhibited by the binding of its UBL domain to its RING1 domain. Activation of parkin is mediated by binding of a ubiquitin molecule that is phosphorylated at Ser_65_ by PINK1 [34]. This binding allows release of the UBL domain, leading to an “open” conformation [34]. To this end, we first docked P-Ub to the auto-inhibited form of our modeled full-length parkin (Fig. 5a, b); this protein-protein docking (P-Ub with parkin) corroborated the previously reported crystal structure data showing P-Ub bound to parkin, specifically to the P-Ub binding helix [34]. This auto-inhibited form of parkin bound to P-Ub was then simulated using a fully solvated all-atom metadynamics approach (Fig. 5c, d). The UBL domain began to move away from the RING1 domain around 30 ns and remained open throughout the simulation (Fig. 5d, Additional file 2: Video S1), providing further evidence for the prevailing hypothesis that P-Ub induces parkin activation by opening up the UBL domain [34]. In addition, this simulated open conformation also gives confidence in our simulation parameters. Metadynamics simulation predicted that as a result of P-Ub binding to parkin, its UBL domain moved 32.2 Å over a 100-ns simulation, exposing Ser_65_ on parkin for phosphorylation, a necessary step in parkin activation (Fig. 5e).

#### NEDD8 activation of parkin

NEDD8 not only translocates from nucleus-to-cytoplasm in Alzheimer brain (Fig. 3), it does so in IL-1β-treated rat primary neuron cultures (Fig. 4). This novel finding of IL-1β induction of NEDD8 translocation is consistent with the idea that neuroinflammation facilitates parkin activation [33], especially in conditions like AD where excesses in IL-1β occur early and continue. Our finding that NEDD8 is structurally similar to ubiquitin, as previously shown in crystal structure, led to us to examine the possibility that NEDD8 acts similarly to P-Ub and allows release of the UBL domain of parkin. Indeed, as we show, NEDD8 does bind to parkin at the same location as P-Ub, i.e., at the P-Ub binding helix (Fig. 5f, g) and, like P-Ub binding, is predicted to expose the Ser_65_ parkin residue by a similar distance (35.6 Å) as that predicted for P-Ub (32.2 Å, Fig. 5h–j, Additional file 3: Video S2). In contrast, simulation of non-phosphorylated ubiquitin is predicted to bind to parkin in a different position from that of P-Ub, missing critical residues from the P-Ub binding helix, specifically residues 311-329 (Additional file 1: Figure S1, highlighted in yellow) [34]. Simulation of parkin with non-phosphorylated ubiquitin did not lead to the UBL-domain release, implying that parkin activation is specific to P-Ub and NEDD8. This being the case, NEDD8 appears to be able to act as a P-Ub substitute that allows for exposure of parkin and its subsequent phosphorylation of Ser_65_. As mentioned above, IL-1β downregulates PINK1 levels, so one might expect, as is the case of the levels of P-Ub in neurons treated with IL-1β, there would be a similar reduction in the levels of P-parkin regardless of NEDD8 or P-Ub binding, and would result in less activation. However, our results show that there is a substantial increase in the levels of P-parkin in IL-1β-treated neurons (Fig. 1g, *p* < 0.001), suggesting that a kinase other than PINK1 can phosphorylate parkin and may do so in conditions, such as those in neurodegenerative diseases, where IL-1β levels are elevated.

### IL-1β increases P-parkin in a GSK3β-dependent manner

**Fig. 6:**
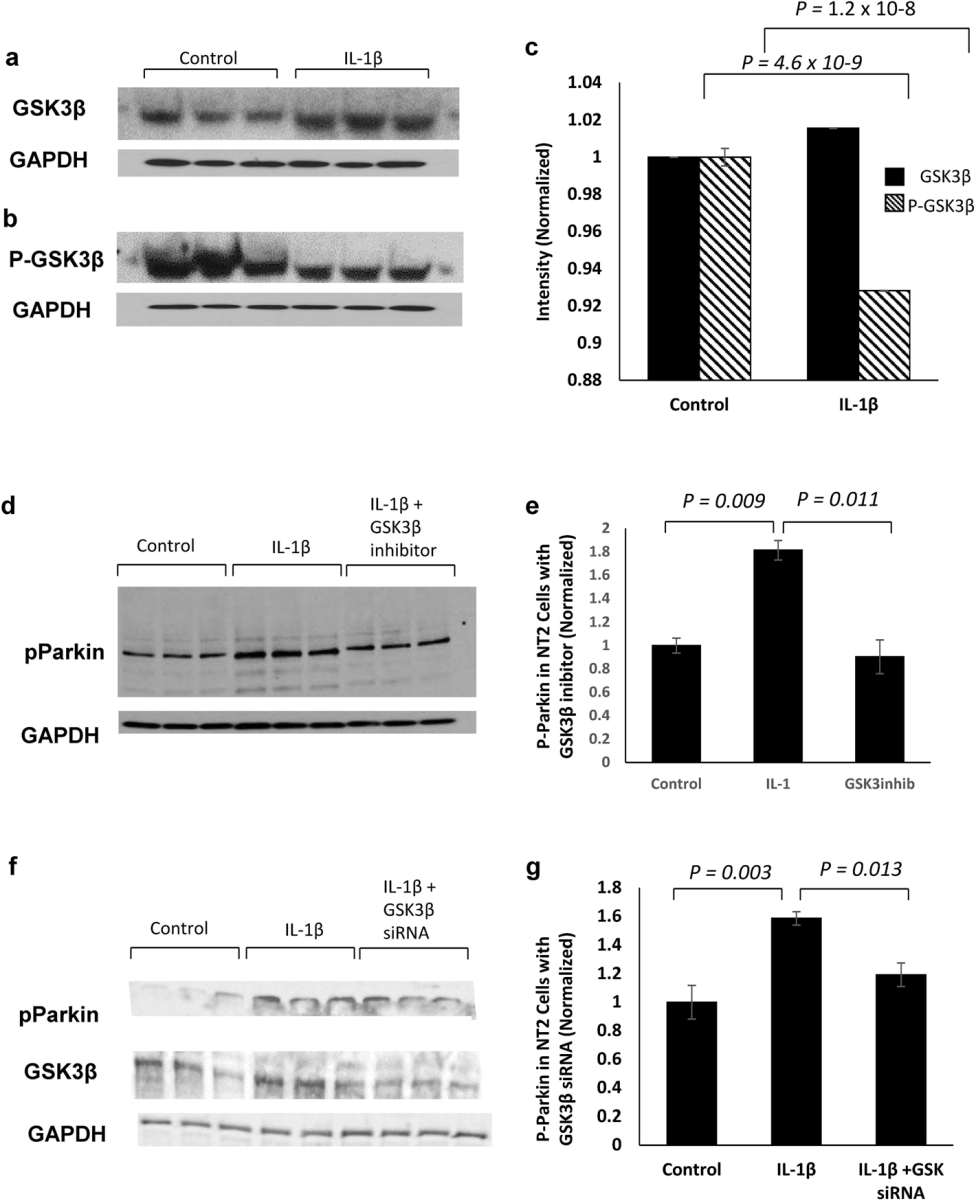
IL-1β increases P-Parkin in a GSK3β-dependent manner. IL-1β-treated NT2 cells have increased levels of GSK3β (**a**, *p* = 4.6 × 10^− 9^) and elevated GSK3β-activation, demonstrated (**b**) via decrease in P-GSK3β (*p* = 1.2 × 10^− 8^) and quantified in (**c**). Western immunoblot analysis of NT2 cells treated with a recognized inhibitor of GSK3β attenuates IL-1β-induced parkin phosphorylation (**d**). Western immunoblot quantification shows a significant decrease in P-parkin levels in NT2 cells treated with GSK3β inhibitor before IL-1β treatment compared with IL-1β treatment alone (*p =* 0.003, one-way ANOVA, significance determined with Bonferroni-corrected *α* = 0.016), quantified in (**e**). siRNA inhibition of GSK3β in NT2 cells attenuated IL-1β-induced parkin phosphorylation (**f**). Quantification of P-parkin levels demonstrates that GSK3β knockdown attenuates IL-1β upregulation of parkin activation (*p =* 0.046, one-way ANOVA, significance determined with Bonferroni-corrected *α* = 0.016) quantified in (**g**)

PINK1 is reported to be the kinase that phosphorylates parkin Ser_65_ as a result of P-Ub binding to parkin and subsequent opening of its UBL domain [22, 31]. As we found that IL-1β treatment of neuronal cultures downregulates PINK1 (Fig. 2), we were surprised to find that P-parkin levels in such cultures were elevated (Fig. 1g), suggesting that a kinase other than PINK1 phosphorylates parkin Ser_65_ when IL-1β is in excess. Based on previous findings suggesting that IL-1β treatment induces GSK3β [10], we measured GSK3β and GSK3β-activation in IL-1β-treated NT2 cells. We confirmed that IL-1β elevates GSK3β levels and decreases P-GSK3β, which is the inactivated form (Fig. 6a–c). We then asked whether GSK3β could increase P-parkin at Ser_65_. To answer this, we treated NT2 cells with known potent GSK3β inhibitors (inhibitors XXVII and AR-A 014418) and showed that IL-1β upregulation of P-parkin was significantly reduced when cultures were pretreated with either of these two specific GSK3β inhibitors (Fig. 6d, e; *p* = 0.003, one-way ANOVA, significance determined with Bonferroni-corrected *α* = 0.016). This indicates that IL-1β, when in excess, mediates elevation of GSK3β, which can lead to phosphorylation parkin at Ser_65_. To further confirm this finding, we treated NT2 cells with IL-1β after siRNA knockdown of the expression of GSK3β and showed a significant reduction in P-parkin levels (Fig. 6f, g, *p* = 0.046, one-way ANOVA, significance determined with Bonferroni-corrected *α* = 0.016), assuring that the IL-1β-induced phosphorylation of parkin occurred despite low levels of PINK1 and at the behest of the IL-1β-mediated increase in the levels of GSK3β.

## Discussion

The presence of excess IL-1β and ubiquitinated aggregates is early and pervasive events in what may be considered as Alzheimer pathogenesis in Down’s syndrome [4, 5] and as we propose, analoguously, in AD [50]. In this study, we tested the hypothesis that there is a compromising interaction between neuroinflammation—noted as excessive levels of IL-1α and β in AD—and the early advent of autophagic failure—noted as the abundance of undigested ubiquitinated intraneuronal aggregates (P-tau) and extracellular aggregates (Aβ) in AD. We further proposed that such may contribute to the neuropathological changes noted in brains of Alzheimer patients. In our studies, we concentrated on providing a more precise understanding of the role of IL-1β in parkin activation, which has been closely related to P-tau and autophagy [12]. Our findings show that (i) parkin is present in P-tau aggregates in neurons lying adjacent to activated IL-1α-laden glia in Alzheimer brain but not in control (AMC) brain; and (ii) the synthesis of IL-1α, IL-1β, and parkin is elevated months prior to the appearance of Aβ plaques in an animal model of AD but not in syngeneic littermates. Further, we found that IL-1β treatment of neurons (iii) increases parkin levels; (iv) reduces the production of PINK1; (v) reduces production of P-Ub, perhaps due to decreased levels of PINK1, thereby decreasing the probability of P-Ub-dependent parkin activation; (vi) induces synthesis of GSK3β; (vii) increases phosphorylation of parkin in a GSK3β-dependent manner; and (viii) induces translocation of NEDD8 from nucleus to cytoplasm, thus, increasing the possibility of NEDD8-parkin interaction. Our simulations predict that both P-Ub and NEDD8 activate parkin by docking at the same site on parkin; and that binding of either P-Ub or NEDD8 to parkin releases the UBL domain and in that way exposes Ser_65_ on parkin for phosphorylation and, thus activation, which results in ubiquitination of unwanted proteins, aggregates, and other compromised cellular entities. Our previous findings regarding neuronal stress-induced upregulation of IL-1α and IL-1β in glia as evidenced by immunoreactive holo-IL-1α inside glia and the presence of IL-1β, resulting from cleavage by IL-1β cleavage enzyme, in medium from sAPPα activated glia and in brain homogenates of patients, together with our finding of an apparent cytological proximity between activated glia overexpressing IL-1α adjacent to neurons having parkin in P-tau-laden aggregates led us to investigate the idea that excess IL-1β released from those highly IL-1α immunopositive glia acts to either stymie or facilitate activation of parkin for ubiquitination of unwanted proteins for lysosomal or proteasomal degradation. Our demonstration of IL-1β downregulation of the levels of PINK1 that may appear simultaneous with upregulation of both the levels and activity of GSK3β and a resultant increase in the levels of P-Parkin is consistent with the idea that the increase in IL-1β in AD may lead to known deleterious effects of GSK3β activation through the IL-1β-mediated decrease in PINK1 but that these may be counterbalanced by NEDD8-dependent activation of parkin via GSK3β activation.

Activated parkin functions to protect neurons from the build-up of spent or misfolded proteins and dysfunctional mitochondria, both of which are characteristic of the neuropathological changes noted in AD and other neurodegenerative diseases. Only two previous studies have reported that parkin is activated by NEDD8; one used coimmunoprecipitation to show endogenous parkin to be neddylated in Parkinson’s disease brain [22], and both showed that the parkin-NEDD8 interaction appears to have elevated ubiquitination activity in vivo and in vitro [22, 33]; although these may be due to neddylation of parkin, our proposed NEDD8-parkin interaction, or a combination of both. In any case, our findings provide strong support for IL-1β as a driver of parkin activation and ubiquitination as follows: (i) IL-1β induces the translocation of NEDD8 from nucleus to cytoplasm in neuronal cells; (ii) NEDD8 and parkin are immediately adjacent in PLA assays; and (iii) our simulation studies predict direct binding of NEDD8 to parkin at the P-Ub site, and that NEDD8, like P-Ub, is predicted to elevate parkin activity via opening of parkin’s auto-inhibitory arm, strongly suggesting that in response to neuronal stress, IL-1β acts to foster NEDD8-related parkin activation. However, as to why this increase in NEDD8-related parkin activation and the resultant readily apparent presence of ubiquitinated aggregates in AD is not sufficient to elicit clearance of such aggregates remains unresolved. In contrast, the lack of evidence of autophagic failure in age-matched patients without AD suggests that it is logical to assume that whatever level of neuronal stress that is tolerable is consonant with tolerable levels of IL-1β in which parkin is sufficiently activated for ubiquitination to meet the challenge of the propensity of excess IL-1β to induce synthesis and activation of kinases that favor untoward phosphorylation of important proteins involved in clearance and in synthesis of proteins important in neuronal repair.

Our simulation results showing that NEDD8 can activate parkin through its direct binding to the P-Ub binding site, together with our observation that NEDD8 is shifted from the nucleus to the cytoplasm in Alzheimer brain and in both NT2 cell and primary rat neuron cultures following treatment with IL-1β, points to a role for IL-1β in parkin-mediated ubiquitination of aggregates. As further support for this role, the mRNAs for, and thus synthesis of IL-1α, IL-1β, and parkin are all elevated months before Aβ plaques appear and increase with age in an AD mouse model. Further, based on our findings that, (i) not only does parkin and NEDD8 interact in AD brain, but, (ii) our finding of IL-1β-induction of NEDD8 translocation from nucleus to cytoplasm, and, (iii) of NEDD8 interaction with and activation of parkin, it is logical to conclude that the AD-associated excess levels of IL-1β play a role in the neuropathologically defined AD-associated parkin pathology. Unfortunately, as is apparent in the aggregate-laden neuropathological manifestations of AD, any felicitous promise of IL-1β toward increasing parkin-mediated ubiquitination and subsequent lysosomal or proteasomal degradation afforded by IL-1β increase is apparently insufficient to overcome the concurrent propensity of IL-1β to promote production of the pathognomonic seed proteins of aggregates in AD and Parkinson’s [10]. In fact, IL-1β further abets this aggregate formation and neuronal stress on normal neuronal tau functions as it increases both the synthesis and activation of the necessary phosphorylating kinases for the production of hyperphosphorylation of tau [7, 13], present in the paired helical filaments in neurofibrillary tangles [51] and α-synuclein in Lewy bodies [10]. In addition, IL-1β creates further neuronal stress as it induces synthesis of the Aβ precursor protein (APP) and increases release of sAPPα for further microglial activation and more synthesis of IL-1β [12]. In addition to the role of excess IL-1β in increasing ubiquitination, sAPPα-mediated excess IL-1β defeats acetylcholine production and release by increasing the synthesis and activity of AChE [14] and by decreasing synaptic density as noted by IL-1β-related decreases in synaptophysin levels in neuronal cell culture and in rats implanted with IL-1β containing slow-release pellets [13].

By way of explanation as to how, or perhaps even why, elevated levels of IL-1β may be beneficial in an acute setting, reparation for acute neuronal stresses could benefit from upregulation of synthesis of a neuron’s acute phase protein APP as such increases in APP are essential for membrane repair, and increases in α-synuclein would favor synaptic integrity and stability, and, as in the present case, parkin activation and consequent ubiquitination are a necessary step in ridding neural cells of unwanted proteins and aggregates. In previous experiments, we have shown that neuronal stress in various forms results in increased expression of the neuronal stress response protein APP and release of sAPPα [52], which in turn activates glia so as to increase synthesis and release of IL-1β [14], which occasions further induction of neuronal APP, and so forth [9, 52]. This cyclic neuronal stress-related activation of IL-1β and APP is known as the Cytokine Cycle [53]. In view of the findings in this report, excess IL-1β influences parkin phosphorylation and activation related to either P-Ub or NEDD8 binding.

## Conclusion

Our results support a role for the elevated expression of IL-1β, noted as an early and continuing event in Alzheimer pathogenesis, in the early steps of autophagy, namely, parkin-mediated ubiquitination of misfolded or otherwise unwanted proteins or organelles. Here we show that in the context of elevated IL-1β: (i) the steady-state levels of parkin and P-parkin are increased; (ii) PINK1 and P-Ub levels are decreased; (iii) NEDD8 is translocated from nucleus-to-cytoplasm in AD and in IL-1β treated neurons; (iv) parkin is activated by binding of NEDD8 at the same location as P-Ub, allowing for exposure of and phosphorylation of parkin Ser_65_; (v) PINK1 levels are reduced, and (vi) P-parkin levels are elevated via IL-1β-induced levels and activation of GSK3β. Taken together, these excess IL-1β-driven events work toward P-parkin-mediated ubiquitination, which has been shown to be neuroprotective in numerous models of neurodegenerative diseases [43]. However, the ubiquitination of undigested aggregates in neurodegenerative diseases characterized by neuroinflammation and elevated IL-1β provides evidence that autophagy is, nevertheless, compromised. This compromise may be due to a combination of excess IL-1β-mediated decreases in PINK1, alongside IL-1β-induced increases in aggregate seed-protein precursors. Our findings, taken together, demonstrate how interactions between two pathology-amending systems—innate immunity and autophagy—can, when gone awry, result in the neuropathological hallmarks of neurodegenerative disease, viz., ubiquitinated aggregates in immature autophagosomes [54].

## Supplementary information

**Additional file 1:** **Figure S1.** Non-phosphorylated ubiquitin (**A**) does not interact with critical residues in the P-Ub binding-site (yellow), and does not lead to opening of the parkin UBL domain, compared to phosphorylated ubiquitin (**B**, and NEDD8, not shown).

**Additional file 2:** **Video S1.** UBL domain opening of parkin with pUB.

**Additional file 3: Video S2.** UBL domain opening of parkin with NEDD8.

## Data Availability

Data sharing is not applicable to this article as no datasets were generated or analyzed during the current study.

## Abbreviations

AD: Alzheimer’s disease
AMC: Age-matched control
APP: Amyloid precursor protein
APP-BP1: Amyloid precursor protein-binding protein 1
Aβ: Amyloid beta
CERAD: Consortium to establish a registry for Alzheimer’s disease
E3 ligase: E3 Ubiquitin ligase
GSK3β: Glycogen synthase kinase-3 beta
IACUC: Institutional Animal Care and Use Committee
IL-1: Interleukin 1
NEDD8: Neural precursor cell -expressed, developmentally downregulated protein 8
PINK1: PTEN-induced putative kinase protein 1
PLA: Proximity ligation assay
PMI: Post-mortem interval
P-parkin: Phosphorylated parkin
P-tau: Phospho-tau
P-Ub: Phosphorylated ubiquitin
RING: Really interesting new gene
RT-PCR: Real-time polymerase chain reaction
sAPPα: Secreted amyloid precursor protein alpha
UBL domain: Ubiquitin-like domain
