# Supplementary figures and images for: Interleukin-1β drives NEDD8 nuclear-to-cytoplasmic translocation, fostering parkin activation via NEDD8 binding to the P-ubiquitin activating site
Source: J Neuroinflammation. 2019 Dec 27;16:275. doi: 10.1186/s12974-019-1669-z (PMC6935243; doi:10.1186/s12974-019-1669-z)

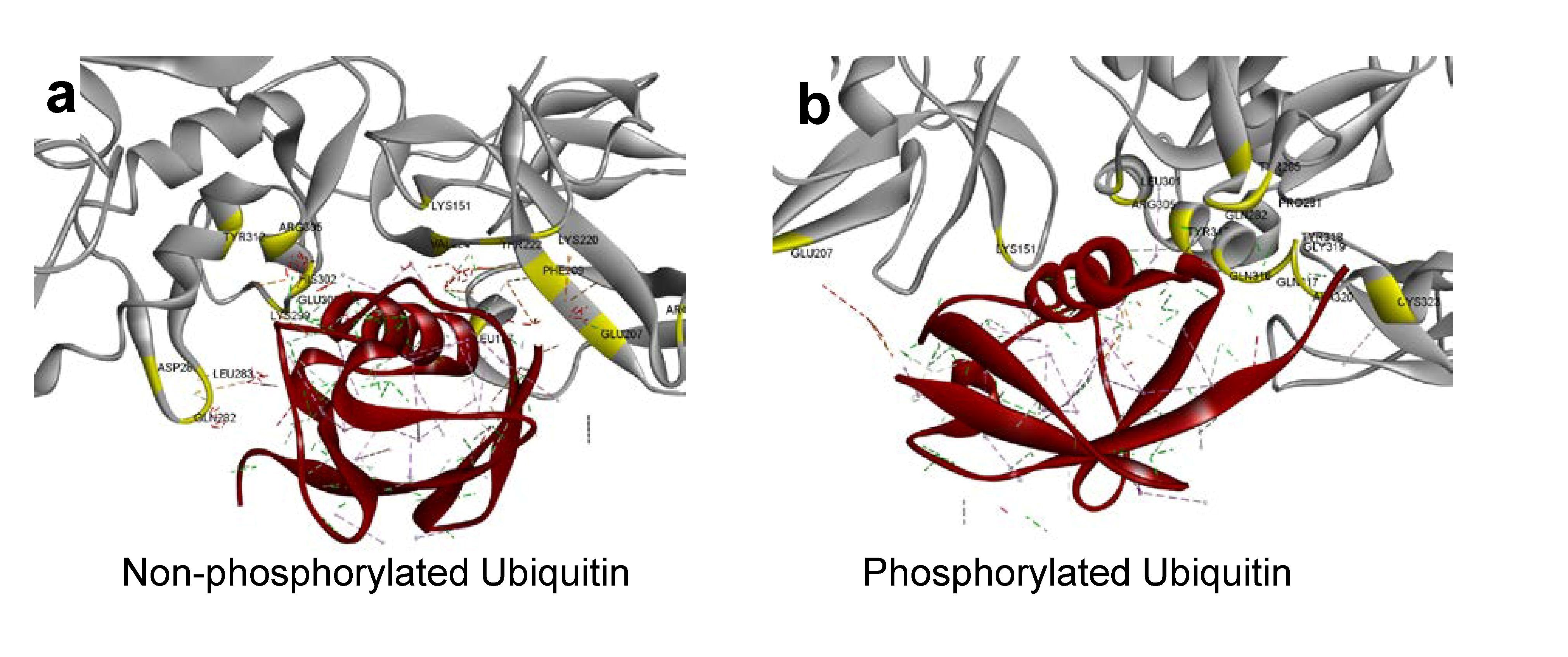

Supplement: Supplementary file 1 — Additional file 1: Figure S1. Non-phosphorylated ubiquitin (A) does not interact with critical residues in the P-Ub binding-site (yellow), and does not lead to opening of the parkin UBL domain, compared to phosphorylated ubiquitin (B, and NEDD8, not shown). [file 12974_2019_1669_MOESM1_ESM.pdf]
